# Removal of failed dental implants revisited: Questions and answers

**DOI:** 10.1002/cre2.234

**Published:** 2019-08-21

**Authors:** Alex Solderer, Adrian Al‐Jazrawi, Philipp Sahrmann, Ronald Jung, Thomas Attin, Patrick R. Schmidlin

**Affiliations:** ^1^ Clinic of Conservative and Preventive Dentistry University of Zurich Zurich Switzerland; ^2^ Private Practice Switzerland; ^3^ Clinic of Reconstructive Dentistry University of Zurich Zurich Switzerland

**Keywords:** dental implants, explantation, failing implant, implant removal

## Abstract

**Objectives:**

This narrative review is aiming on showing reasons for implant failure, removal techniques, and respective clinical considerations; further, the survival rate of implants in previous failed sites is examined.

**Materials and methods:**

Questions have been formulated, answered, and discussed through a literature search including studies assessing implant failure and removal up to 2018.

**Results:**

Studies describing reasons for implant failure, implant removal techniques, and the reinsertion of implants in a previous failed site (*n* = 12) were included. To date, peri‐implantitis is the main reason for late implant failure (81.9%). Trephine burs seem to be the best‐known method for implant removal. Nevertheless, the counter‐torque‐ratchet‐technique, because of the low invasiveness, should be the first choice for the clinician. Regarding zirconia implant removal, only scarce data are available. Implantation in previously failed sites irrespective of an early or late failure results in 71% to 100% survival over 5 years.

**Conclusion:**

If removal is required, interventions should be based on considerations regarding minimally invasive access and management as well as predictable healing. (Post)Operative considerations should primarily depend on the defect type and the consecutive implantation plans.

## 1 INTRODUCTION

Periodontitis and dental caries are the main causes for tooth loss. The prevalence of (partially) edentulous patients worldwide varies between 7% and 69% (Petersen, Bourgeois, Bratthall, & Ogawa, 2005). Removable and fixed prostheses have been—and still are—used to restore masticatory function and esthetics. Dental implants, however, have become a great additional treatment option to replace missing teeth, and respective treatment concepts have reported high success rates of 97% and 75% over 10 (Buser et al., 2012) and 20 years, (Chappuis et al., 2013) respectively. Nevertheless, like in every medical therapeutic intervention, biological complications occur, which may finally lead to complete implant failure and consequently—in the worst case—to the removal of the implant.

In general, implant failures can be described as early or late events in terms of time‐point characterization. These definitions are mainly based on initial healing and restorative stages. Whereas early failures occur before the implants are functionally loaded and therefore mainly represent an inadequate healing and osseointegration at the initial stage, late failures are observed after loading and function. Early implant failures can have multiple causes, that is, overheating of the bone during preparation of the implant site, lack of primary stability due to overpreparation of the implant site or poor bone quality, overload, or parafunctions (Froum et al., 2011). In this context, implants are clinically mobile and therefore easy to remove. In contrast, late implant failures are mainly due to biological reasons. Bone loss due to peri‐implantitis or implant fractures are the most prevalent ones. In very rare cases, even healthy and osseointegrated implants may be regarded as failures due to extreme malpositioning and therefore prosthetic reasons. In this situation, implant removal might be considered as well.

Most late‐failing implants are not mobile and remain at least partially osseointegrated in the very apical aspect. The attempt to remove the implants can therefore still be very challenging, and the explantation may be invasive, and neighbor teeth and structures can also be potentially harmed (Froum et al., 2011). Not surprisingly different methods of implant removal have been described in the literature so far including the use of counter‐torque ratchet (2016), piezo surgery (2002), high‐speed burs, elevators, forceps, trephine burs (2018)(2016) and laser surgery (2016). (Bowkett, Laverty, Patel, & Addy, 2016) counter‐torque ratchet, (Simon & Caputo, 2002) piezo surgery, (Messina, Marini, & Marini, 2018) high‐speed burs, elevators, forceps, trephine burs (Deeb, Koerich, Whitley, & Bencharit, 2018), (Bowkett et al., 2016) and laser surgery (Hajji et al., 2016).

Whereas a plethora of reviews and studies are dealing with general implantologic topics focusing on (pre‐) surgical and (pre‐)prosthetic aspects of implants in health and disease, respective literature on the potential advantages and disadvantages of different removal techniques are still scarce. The last update on removal techniques was given in 2016 (Bowkett et al., 2016). Therefore, this overview aimed to outline indications for implant removal and to put the various techniques into a clinical context of today. Further it is the very first review where considerations of ailing and failing zirconia implants are assessed and summarized. In a narrative review, clinically relevant questions were formulated, answered and discussed based on the available literature.

## 2 MATERIALS AND METHODS

A literature search was done for studies and articles assessing the removal of dental implants including etiology and techniques for implant removal. Main focus was to collect data from the published literature on implant removal in general addressing the following specific questions:

1. When do implants need to be removed?
2. How should dental implants get removed?
3. How does the implant material influence the removal approach?
4. What is the effect of implant removal on the surrounding bone? Does the explanation site require specific socket preparation?
5. What should be considered when reinserting implants?

The following databases were included: PubMed, MEDLINE, Embase, and Cochrane library. And the following MeSH terms were searched:

- Population: patients with dental implants.
- Health condition: Failed OR Failing OR ailing OR Fracture OR Malpositioned OR Periimplantitis.
- Therapy: Removal OR Explantation OR Explant re‐treat OR retreat OR redo OR reoperate OR re‐operate OR previously failed OR replant OR re‐plant OR reimplant OR re‐implant OR reinsert OR “re‐insert.”

## 3 SCREENING AND SELECTION

Two authors (A. S. and A. A.) independently assessed the publications by title and abstract. The inclusion and exclusion criteria for the studies were as follows:

1.Inclusion criteria: RCTs (Randomized Clinical Trials) and case series, retrospective studies, case reports, systematic reviews, and narrative reviews.

2.Exclusion criteria: not osseointegrated implants, no screw‐shaped implants, implants used in orthodontics, and animal studies.

Available titles and abstracts were collected and discussed before being finally included or excluded. Interexaminer agreement of a Cohen's kappa (*K*) of 0.65 was achieved after initial screening. Authors discussed discrepancies until reaching consent. If required, the senior author (PRS) was consulted. Out of 3,997 screened articles, finally, 34 studies were included.

Table 1 shows the summary of evidence regarding Questions 1–4, whereas Table 2 is providing an overview of the studies assessing Question 5.

**Table 1:** Summary of evidence assessing Questions 1–4

Author/year	Topic of interest	Study type	No. of implants evaluated	Conclusion
Anitua, Murias‐Freijo, and Alkhraisat (2016)	Explantation—CTRT	Case series	91	Extraction torques range from 80 to 200 Ncm Exceeding torques need a 2‐ to 3‐mm deep cut with a trephine bur
Anitua, Piñas, Begoña, and Alkhraisat (2017)	Implant failure	Retrospective Pilot‐Study	158	Peri‐implantitis ➔ main reason for implant failure (82.9%)
Chrcanovic, Kisch, Albrektsson, and Wennerberg (2017)	Implant failure	Retrospective	10.096	6.36% of implants fail 49% of failures in the first year
Cunliffe and Barclay (2011)	Explantation—electrosurgery	Case report	1	More research is needed
Deeb et al. (2018)	Explantation—trephine burs	Case report	3	3D‐guided use of trephine burs might be less invasive
Derks and Tomasi (2015)	Peri‐implantitis	Meta‐analysis	1.556	22% of all implants develop peri‐implantitis
Froum et al. (2011)	Explantation—review	Review	—	Counter‐torque ratchet technique/reverse screw technique ➔ least invasive method
Greenstein and Cavallaro (2014)	Implant failure	Review	—	More than 75% bone loss defines failure
Lee, Kim, Jeong, Kim, and Lee (2018)	Implant fracture	Retrospective	19.087	0.4% of implants fracture
Messina et al. (2018)	Explantation—piezo surgery	Case series	10 patients	Less invasive compared with trephine burs
Misch et al. (2008)	Implant failure	Review	—	More than 50% bone loss defines failure
Nishihara, Haro Adanez, and Att (2018)	Zirconia implants	In vitro	—	Removal torque of zircona and titanium implants are similar
Schlichting, Padture, and Klemens (2001)	Zirconia implants	In vitro	—	Fracture resistance in titanium implants is much higher
Schwarz et al. (2007)	Peri‐implantitis defects	In vivo	40 in humans and 15 in dogs	Circumferential defects are most common in humans and dogs (55.3%)
Sendyk, Chrcanovic, Albrektsson, Wennerberg, and Zindel Deboni (2017)	Surgical malpositioning	Meta‐analysis	Exp. 37.695 Inexp. 5.901	Experienced surgeons: 2.45% failure Inexperienced surgeons: 12.2% failure
Smith and Rose (2010)	Explantation—laser surgery	Case report	1	Laser: less invasive More time consuming

**Table 2:** Studies assessing survival and success of implants placed in previously failed sites

Author	No. of patients/implants	Follow‐up	Reason of failure	Survival/success rate of implant after implant removal	Survival/success rate of third attempt (no. of failed)
Raghoebar, Meijer, van Minnen, and Vissink (2018)	16/16	12 months	Peri‐implantitis	Survival and success: 100%	—
Anitua et al. (2017)	17/22	9–52 months	Peri‐implantitis	Survival: 94.7%	—
Chrcanovic et al. (2017)	98/175	—	—	Survival: 73%	Survival 64.3% (5/14)
Manor, Chaushu, Lorean, and Mijiritzky (2015)	75/75 Test: in grafted maxillary sinus(Lang & Lindhe, 2015) Control: in nongrafted maxilla (35)	17.6–133 months (avg. 58.4)	Biological complications Early failure Failed Osseointegration in 77.3%	Survival T: 100% C: 92%	—
Wang et al. (2015)	66/67	Avg. 69.4 months	Early failure Failed Osseointegration	Success: 90.6% Survival: 94.6%	—
Quaranta, Perrotti, Piattelli, Piemontese, and Procaccini (2014)	10/16	36 months	Early failure Failed Osseointegration	Survival: 100% Success: 93.75%	—
Mardinger, Ben Zvi, Chaushu, Nissan, and Manor (2012)	144/144	12–180 months	Mixed	Survival: 93%	85% (1/7)
Kim, Park, Kim, and Lee (2010)	49/60	7–36 months	—	Survival: 88.7%	100% (0/7)
(Grossmann & Levin, 2007)	75/96	6–64 months	—	Survival: 71%	50% (1/2)
(Machtei, Horwitz, Mahler, Grossmann, & Levin, 2011; Machtei, Mahler, Oettinger‐Barak, Zuabi, & Horwitz, 2008)	56/79	7–78 months	Mixed	Survival: 83.5%	Survival: 60% (6/15)
Alsaadi, Quirynen, & van Steenberghe, 2006)	41/58 29 machined surface 19 TiUnite replaced w. machined surface 10 TiUnite replaced TiUnite	9–49 months	—	Survival:79.3% 95% 100%	—
Covani, Barone, Cornelini, & Crespi, 2006)	9/9	12 months	Mechanical fracture	Survival and success: 100%	—

## 4 QUESTION 1: WHEN AND WHY DO DENTAL IMPLANTS NEED TO BE REMOVED?

**Figure 1:**
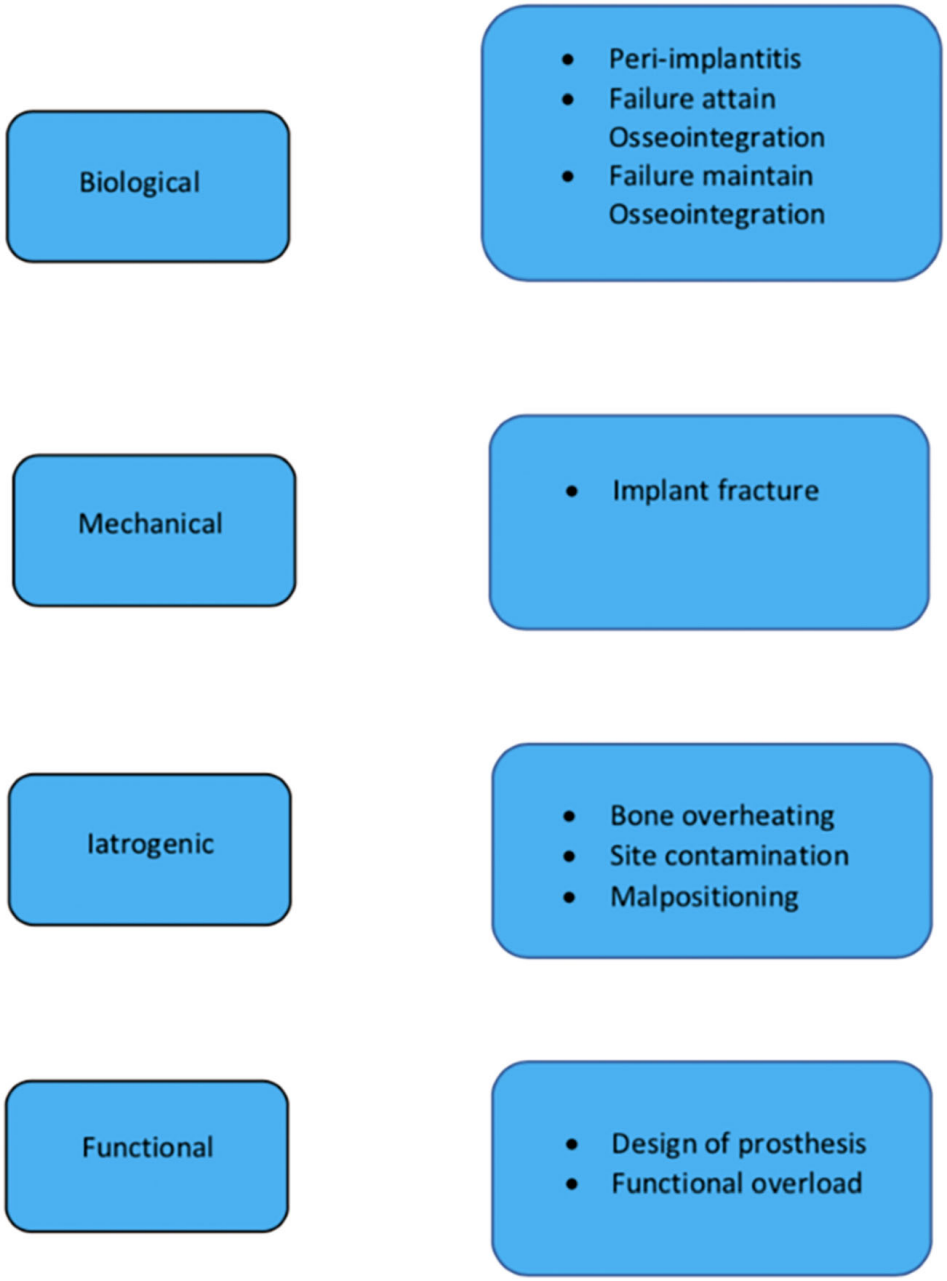
Classification of etiologic reasons for an implant failure (11)

In some cases, as mentioned already above, dental implants fail and need to be removed due to different reasons (Figure 1). Chrcanovic and co‐workers analyzed 10.096 implants, of which 642 were recorded as failures (6.36%). Forty‐nine percent of all reported failures and removals were diagnosed and/or took place during the first year after surgery (Chrcanovic et al., 2017). In summary, the etiologic reasons for failures can be categorized as being of biological, mechanical, iatrogenic, and/or functional origin (Esposito, Thomsen, Ericson, Sennerby, & Lekholm, 2000). Figure 1 provides an overview over the etiology of implant failures including early and late failures:

- Early failure: failure to attain or maintain osseointegration, bone overheating, site contamination. These implants are normally mobile and easy to remove.
- Late implant failure: progressive peri‐implantitis, implant fractures, and malpositioned implants. These implants are more difficult to remove due to an at least partly osseointegrated implant proportion.

### 4.1 Biological failures

Peri‐implantitis represents the major biological complication and is described as a pathological condition occurring in tissues around functionally loaded dental implants, characterized by inflammation of the mucosa and progressive marginal bone loss (Schwarz, Derks, Monje, & Wang, 2018). It represents the main reason for late implant failure (Anitua et al., 2017; Manor et al., 2015). Derks et al. (2015) reported in a meta‐analysis that 22% of all implants will develop peri‐implantitis. (Anitua et al., 2017) assessed 81 patients with 158 non‐mobile implants, which were scheduled for explantation. The main reason for implant removal was peri‐implantitis (82.9%), followed by malpositioning in 13.9% of the cases. Again, peri‐implantitis was generally considered being the main reason for late implant failure. Clinical symptoms around implants with peri‐implantitis are quite similar to those we can find around periodontally affected teeth, which include bleeding upon gentle probing of the adjacent gingiva/mucosa, pain, suppuration, increased probing depth, radiographic bone loss, and the presence of pathogenic bacteria, which colonize nonshedding surfaces colonizing biofilms. In contrast to teeth, implants will not become mobile, unless—in a final stage—osseointegration is more or less completely lost.

Whereas dental implants with advanced biological bone loss can be treated, there is still some controversy regarding the indications and prognoses of the described nonsurgical and surgical therapeutic approaches and their outcomes. However, implant removal can always be a valuable (last) therapeutic option, especially in progressed cases. Taking the decision, whether to treat or to remove a partially osseointegrated implant, remains always challenging, and clear guidelines are still missing and therefore warranted. Whereas some studies defined an implant with more than 50% bone loss as a failure, (Misch et al., 2008) other authors propose ≥75% of bone loss or less as 3 mm of remaining bone contact as a critical threshold (Misch et al., 2008). Because any guideline depends on the length of an individual affected implant, factors such as progression, patient comfort, elimination of risk factors, and prosthetic treatment options, decision making remains a sophisticated issue (Greenstein & Cavallaro, 2014).

### 4.2 Mechanical failures

Implant fractures have been described; possible causes include bruxism, large occlusal forces, mechanical trauma, reduced implant diameters, material fatigue, and advanced bone loss leading to reduced mechanical support around the implant. Risk of fracture is increasing over lifetime of the implant (Sanivarapu, Moogla, Kuntcham, & Kolaparthy, 2016). (Goodacre, Bernal, Rungcharassaeng, & Kan, 2003) described the risk of implant fractures with a quite low prevalence of 1%. A risk seems to exist especially in the molar region (Misch & Degidi, 2003). Implantoplasty is a widespread method of peri‐implantitis treatment. Hereby, implant threads are smoothened and polished in order to get a less plaque‐accumulative surface (Romeo et al., 2005).

More recent data concerning fractures was published by (Lee et al., 2018) assessing 19.087 implants in 8,501 patients. Fractures were observed in 70 implants (0.4%) and 57 patients.

With regard to zirconia implants, less data are available. Roehling and co‐workers assessed in a systematic review the fracture rate of zirconia implants among other parameters. In three studies with a total of 275 implants, a slightly higher overall fracture rate of 1.95% (22 implants) was described. Noteworthy, not commercially available implants were also included in this data set. Focusing exclusively on implants available on the market, the fracture rate decreased again to 0.2%. Bearing the limited data available in mind, these results, however, seem to indicate a similar fracture rate of zirconia and titanium implants.

### 4.3 Surgical malpositioning

An incorrect position of the implant (location, inclination, etc.) may impede an adequate prosthetic rehabilitation in many cases. Such problems are mainly caused by poor treatment planning or an inaccurate surgical execution. Approximately 10% of all implants show a prosthetically inadequate position, meaning that these implants were not even able to be adequately loaded (Becktor, Isaksson, & Sennerby, 2004). As a result, biomechanical problems due to wrong occlusal force axis, an inacceptable aesthetic appearance, or difficulties in maintaining proper hygiene may be the consequence (Chee & Jivraj, 2007). Therefore, this situation may correctly be considered as failure as well, which may require explantation. In 2017, a meta‐analysis assessed the impact of surgical experience of the dentist on implant failure rates (Sendyk et al., 2017). It was found that implants placed by experienced surgeons (over 50 implants per year) showed a failure rate of 2.4% (out of 85 implants), whereas implants placed by dentists and surgeons with less routine (under 50 implants per year) had a failure rate of 12.2%. The resulting odds ratio (OR) for failure was 2.18 (95% CI [1.40, 3.39]) for less experienced clinicians.

## 5 QUESTION 2: HOW SHOULD DENTAL IMPLANTS BE REMOVED?

Once the decision for the removal of an implant has been made, the selection of the appropriate removal technique should be addressed. The selected option should be fast, as minimally traumatic as possible and cost‐effective for patient and dentist. With regard to any qualitative or quantitative evaluation regarding these aspects, only case reports or series have been published so far according to the best of the author's knowledge (Table 1). Below, the basic techniques are summarized according to the available literature.

### 5.1 Tooth extraction set

Dental implants, which are mobile or show only little residual bone‐to‐implant contact, can usually be removed with instruments, which are also used for tooth‐extraction including levers, elevators, and/or forceps. If the threads oppose no resistance, rotating movements are not even required.

### 5.2 Trephine burs

Most trephine burs are characterized by cylindrical blades. They are widely considered as a standard approach and therefore still represent a very common method to remove implants. These burs exist in different diameters and should be chosen being only little broader than the actual implant diameter in order to remove as less as possible of the remaining bone. A cutting speed ranging between 1,200 and 1,500 rpm is recommended with maximal water cooling in order to avoid any overheating and thermal necrosis (Froum et al., 2011). The trephine burs should be used, however, only if no less invasive alternative techniques are applicable. Because complications as fractures of the mandible and osteomyelitis have been described in case reports, the local anatomy should be carefully assessed including conventional and—if needed—cone‐beam radiology (Bowkett et al., 2016).

A new approach in trephine bur removal was recently described by Deeb et al. (2018): They described the use of a CAD/CAM generated surgical guides, which can be used for guided explantation as well. The authors concluded that 3D guided might allow a more accurate and less invasive surgery.

### 5.3 Piezo surgery

There are few case series and case reports (Messina et al., 2018) assessing this technique. As compared with trephine burs, this method allows for a less traumatic surgical approach for the removal of the failed implants. The authors point out the fact that often blood vessels and nerves can be found in close proximity to implants, which can be harmed. The devices operate at frequencies ranging from 24.000 to 29.500 Hz, which apparently allows for a precise and selective cutting in order to conserve sensitive structures (Messina et al., 2018).

Basically, a circumferential osteotomy is done with a diamond‐coated insert attached to a piezoelectric device. Thereby, the implant‐bone interface is destroyed by ultrasonic waves; an intermitting application mode and proper cooling with saline solution is, however, also mandatory. The osteotomy is performed as close as possible to the implant surface in order to remove only the least necessary amount of bone. The method was mostly described in combination with fractured and malpositioned implants. Improved postoperative bone healing compared with trephine bur surgery was observed (Froum et al., 2011).

Noteworthy, caution has to be taken, when piezo surgery is applied in patients with pacemakers. Although an in vitro study showed no respective side effects, further evidence is still needed (Gomez, Jara, Sánchez, Roig, & Duran‐Sindreu, 2013).

### 5.4 Laser surgery

One case report described the removal of a single dental implant using an Er,Cr:YSGG‐laser (Smith & Rose, 2010). The procedure was described as being similar to piezo‐surgical interventions, because a circumferential destruction of the bone‐implant interface by the laser device is achieved. The laser generates pulsed photons, which absorbed by water leading to microexplosions and destruction on the surrounding target tissue. This procedure is described as the hydrokinetic effect and leads to clean cuts without any thermal damage. As stipulated by the authors, (Smith & Rose, 2010) the method should potentially be less invasive as compared with other techniques. As a further advantage of laser surgery, an optimal hemostatic control was reported, thus facilitating good visualization and therefore accelerating the intervention.

Another in vitro study on human mandibles used the same laser and compared it to the conventional trephine approach.(Hajji et al., 2016) Assessed parameters were the amount of the removed bone, duration of the procedure, and morphological alterations on the surface of the bone. The procedures were conducted on six implants in each group (length: 12 mm; diameter: 5 mm). The results showed almost half of removed bone through the laser compared with the trephine burs (0.302 vs. 0.519 cm^3^). Regarding the duration of the procedure, the trephine burs were more than twice as fast as the laser (17.2 vs. 44.1 s). Assessing morphological bone alterations in the laser group, the authors found well‐defined bone edges without any thermal alteration, whereas the trephine bur group showed abnormal bone formations with some microcracks.

In conclusion, laser surgery showed a less invasive and traumatic intervention as compared with trephine burs; however, the procedure was more time consuming. Although laser surgery is already established in the fields of periodontology and implant dentistry (Aoki et al., 2015) and some preliminary data seem promising, more research is needed before this technique can be recommended in general.

### 5.5 Counter‐torque ratchet technique

The counter‐torque ratchet technique (CTRT) is reported to represent the least traumatic technique in order to remove failed implants (Froum et al., 2011). The application of this method allows to keep the surrounding bone more or less undamaged.

Two different CTRT modalities have been described so far: The first option requires an intact implant connection in order to loosen the fixture. Hereby, a fitting abutment or engaging extraction tool is placed in or on the implant. The removal is done through a counterclockwise torque. Different factors affecting this technique are described. First, due to a higher leverage, an implant with an internal connection is easier to be removed than implants with external connections. Second, the different implant thread shapes, namely, buttress, square, V‐shaped, and reverse buttress may influence the removal, because of the different bone‐to‐implant contact. Square threads are described to have the highest bone‐to‐implant contact and are therefore harder to remove. Next, the implant body design is affecting an implant removal. Tapered implants are reported to be removed easier than parallel ones. Finally, the antirotational design of some implants especially found in the apical region might hamper the unwinding as well (Misch & Resnik, 2017).

The second option is the reverse screw technique (RST), which finds its application mainly in the removal of fractured and damaged implants. In the latter, a screw is driven counterclockwise into the damaged implant in order to get grip in the damaged implant. Afterwards, counter‐torque‐wise force is applied to remove the unit as a whole (Froum et al., 2011). Force is applied until the resistance drops, and the implant can be easily unscrewed without force. Some authors also recommend to cool the bone with saline during this first unscrewing phase stating that the high friction might increases the bone temperature (Stajcic et al., 2016).

Simon and Caputo (Simon & Caputo, 2002) evaluated a counter‐torque method to remove transitional orthodontic implants. Thirty‐one 1.8‐mm diameter orthodontic mini implants were removed using a modified ITI (ITI‐Straumann) torque driver employing CTRT. Twenty‐six of these implants were removed intact with a torque ranging between 11 and 23 Ncm. The remaining implants fractured at the bone level at torques between 27 and 35 Ncm and could not get removed by CTRT. Anitua et al. (Anitua & Orive, 2012) described the counter‐torque method using the BTI explantation kit (BTI Biotechnology Institute, Vitoria, Spain) for osseointegrated implants. Again, the goal of this technique is again to remove the implant as atraumatic as possible in order to ensure the possibility of a second implantation best possible. The authors assessed 42 patients with a total of 91 implants. Seventy‐eight implants were removed only by CTRT, whereas 13 implants still needed the combination of trephine burs and the BTI system. Another trial with 81 patients and 158 nonmobile implants, which were scheduled for explantation, showed that 139 implants were removed with a torque of 146 Ncm without adjunctive use of burs (Anitua et al., 2016). Again, 19 interventions trephine burs and a higher torque of 161 Ncm had to be used. Indication for the use of trephine burs were initial removal torques higher than 200 Ncm, fractured implants and fractured prosthetic components. The removal torque was statistically significantly lower in plasma‐sprayed implants than in other surfaces. The highest torque was found for the removal of acid etched and sand‐blasted implants.

**Figure 2:**
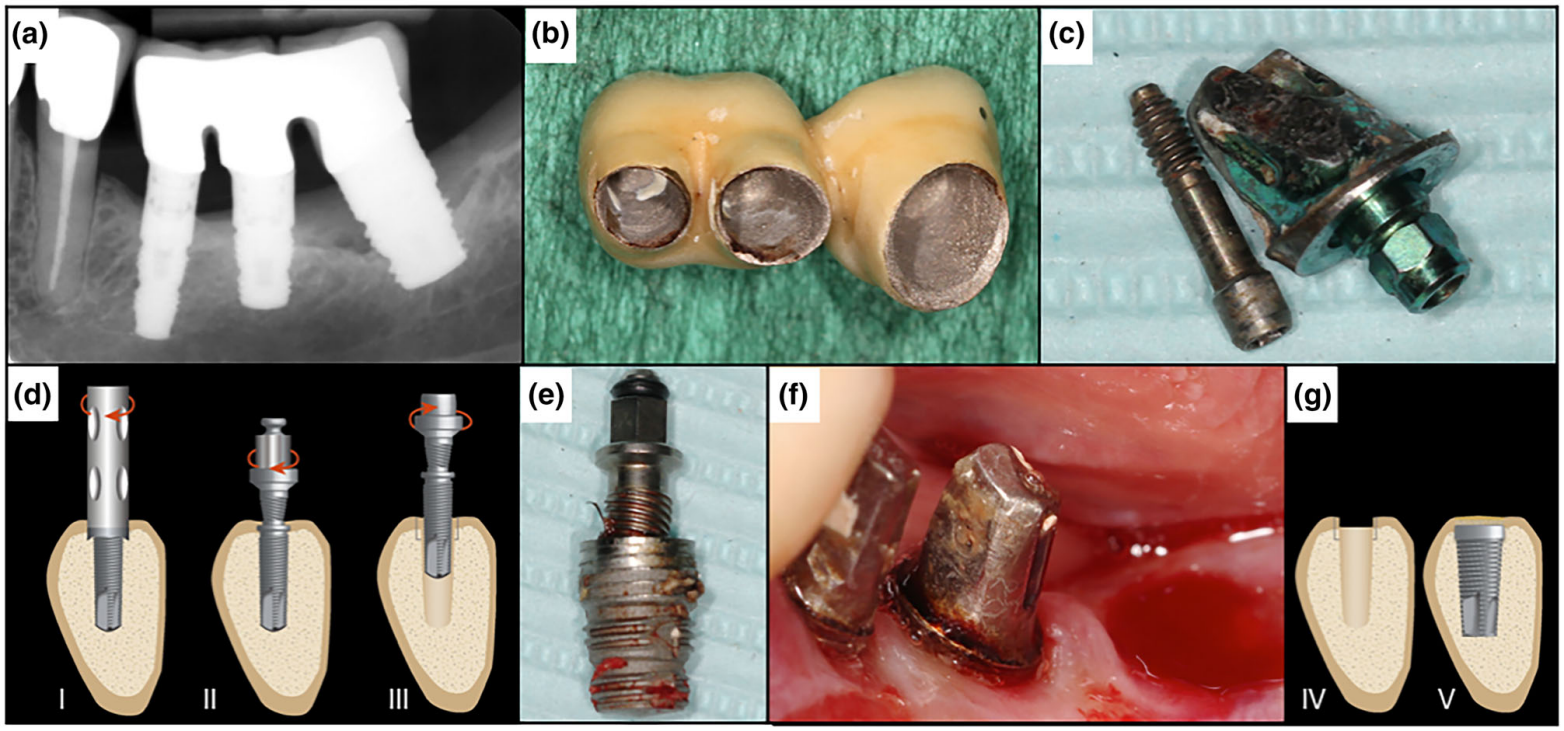
Illustration showing the reverse screw technique. (a) Preoperative X‐ray showing advanced peri‐implantitis in Region 38. (b) Removal of the temporary cemented three‐piece bridge. (c) Disconnection of the abutment. (d) (I) If needed, a trephine bur is used to remove the first 2 cm of bone‐to‐implant contact (in this case not needed); (II) the screw is applied and cut counterclockwise into the implant; (III) counterclockwise torque is applied until the implant becomes loose and is unwinded. (e) Removed implant from Region 38. (f) Post‐operation site. (g) (IV) The socket has been kept in good condition and ready for regeneration and/or for a new implant (V) (as described by Anitua and Orive, 2012)

**Figure 3:**
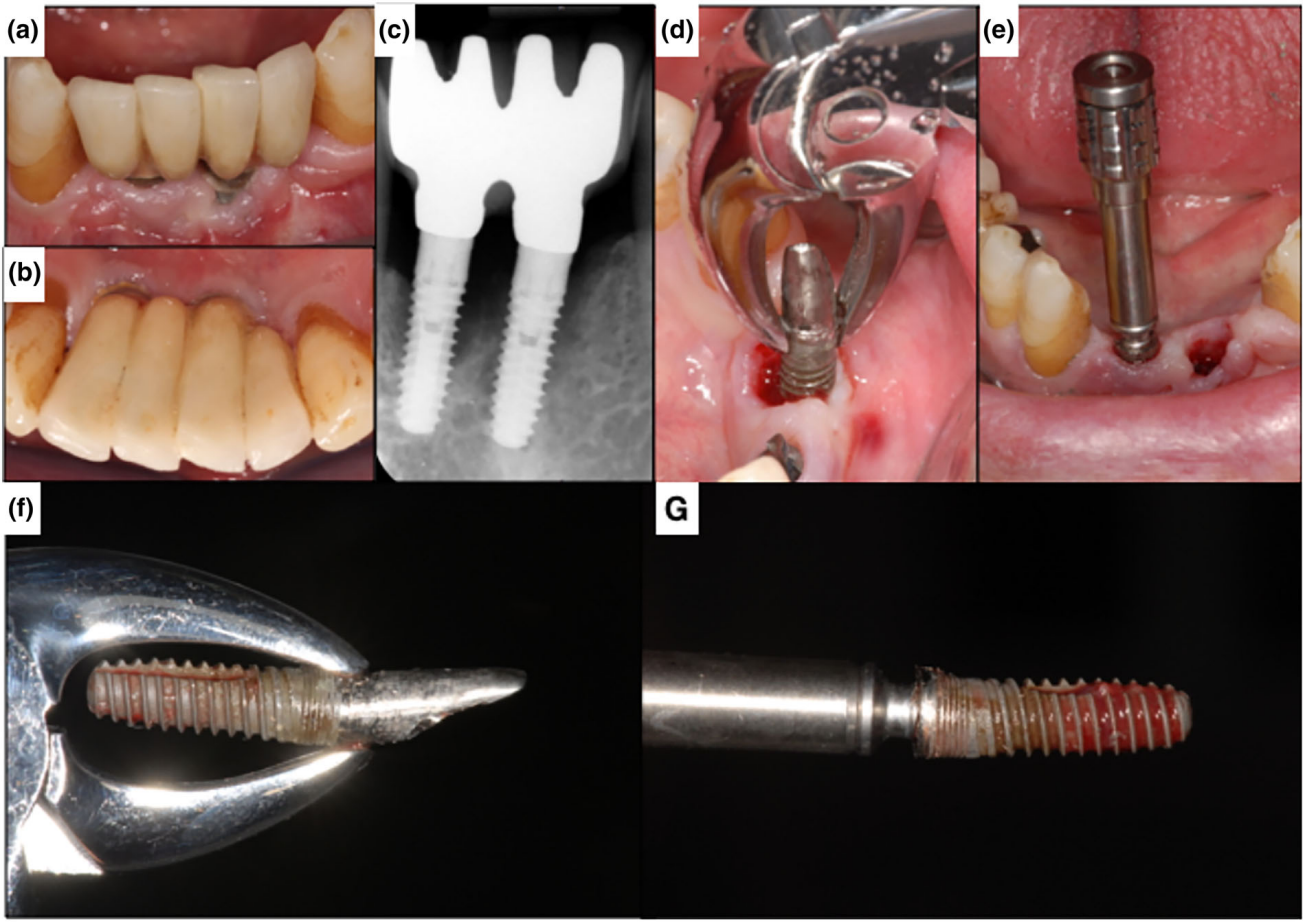
Clinical case with a combined approach using a forceps (d) (minute residual bone) and the reverse screw technique (e). (a,b) Preoperative clinical situation. (c) X‐ray. Arrows showing the bone defect depth (d). Removal of the implant (c,f) with a forceps by counterclockwise rotation. (e) Implant removal (c,g) with the reverse screw technique. Arrows showing height of previous bone level. (f,g) Showing both implants after removal. Arrows showing height of previous bone level

Four millimeters of remaining osseointegration was described as a critical number for the decision of using the CTRT method alone. Implants with more osseointegration are recommended to be removed with a combination of CTRT and a bone‐cutting method, as trephine burs or piezo (Balaji & Balaji, 2018). Figures 2 and 3 show clinical cases of implant removal with the reverse screw technique as recommended by Anitua et al. (Anitua & Orive, 2012). In any case, clinicians should be aware of possible implant fractures, especially in narrow implants (Froum et al., 2011).

Several CTRT sets do exist meanwhile on the market.

### 5.6 Electrosurgery

The idea behind this approach is to cause a distinct thermo‐necrosis at the bone‐implant interface in order to be able to remove the implant at a low counterclockwise torque after osseodisintegration aiming to be as mechanically atraumatic as possible. Osteonecrosis due to thermal reasons is a condition that results in local bone death by loss of blood supply and primary or secondary death of bone cells (Augustin et al., 2008). In one study describing the respective technique, the authors applied an ultra‐high frequency mono‐polar electrosurgery unit for 15 s in a malpositioned implant (Cunliffe & Barclay, 2011). One week later, the implant could be removed with a counter torque ratchet at 30 N. The main concern of the authors with this technique was the development of mucosal and extended osteonecrosis. In the literature, temperatures above 56°C to 70°C are considered harmful to bone tissues, mainly also because of the transformation of alkaline phosphatase (Berman, Reid, Yanicko, Sih, & Zimmerman, 1984). Eriksson and Albrektsson (Eriksson & Albrektsson, 1984) reported in their studies that bone heated to a temperature ranging from 44°C to 47°C for over 1 min would already cause thermal necrosis. A more recent study reported 47°C as critical temperature for the development of thermal osteonecrosis in bone. Osteonecrosis due to thermal reasons is a condition that result in local bone death by loss of blood supply and death of bone cells (Augustin et al., 2008). Despite being promising and anecdotally reported and performed by some dentists (also by heating implants using blunt diamond burs without water supply), clearly more research has to be done in this field to allow for a reliable clinical justification of this approach (Cunliffe & Barclay, 2011).

## 6 QUESTION 3: HOW DOES IMPLANT MATERIAL INFLUENCE IMPLANT REMOVAL?

Zirconia's overall favorable mechanical and esthetic properties, a claimed lower susceptibility to plaque formation, and last but not least an excellent biocompatibility are encouraging arguments for its use in modern implant dentistry. In terms of osseointegration, zirconia implants have been shown to have a similar performance as compared with titanium implants (Nishihara et al., 2018). In this context, a systematic review showed bone‐to‐implant contacts of zirconia and titanium implants ranging from 25 to 88% and 24–85%, respectively. In addition, the removal torque (RT) seems to be comparable as well, ranging from 9 to 78 N for zirconia and 7–74 N for titanium implants (Nishihara et al., 2018). However, many other questions regarding zirconia implants remain still unanswered so far (Nishihara et al., 2018). For example, there is a lack of information in the literature regarding the removal of failed osseointegrated zirconia implants by mechanical or other means. The potential fracture susceptibility and different temperature transduction ranges force one to take a closer look at some basic information about zirconia itself in order to indirectly answer this question.

Most probably—with regard to the resection of adjacent bone as such, the material properties do not play a major role. Dental extraction kit, trephine burs, piezo, and laser surgery can potentially be used in a comparable manner. However, when it comes to CTRT—due to the reduced fracture toughness—more caution may be required.

Whereas zirconia implants show fracture toughness values ranging from 4 to 18 MPa/m, (Nishihara et al., 2018) titanium is much higher 77 MPa/m. (Schlichting et al., 2001) Therefore, it seems more likely that zirconia implants are not eligible to be removed with the CTRT, as they would probably fracture. Only in cases, where the surrounding bone has suffered an extensive resorption process, the minimal‐invasive CTRT might be an option. However, no data are available on this topic so far.

Regarding a possible electro surgical approach to remove a zirconium implant, no data exist as well. Even data on thermal conductivity of zirconia‐based dental implant materials are missing. As in literature Zirconia is described even as a thermal isolator at elevated temperatures, it hints to the fact that also this method may not be appropriate for zirconia implants (Schlichting et al., 2001).

## 7 QUESTION 4: WHAT IS THE FATE OF THE SURROUNDING BONE AFTER IMPLANT REMOVAL? DO EXPLANATION SITES REQUIRE A SPECIFIC SOCKET PREPARATION?

Compared with natural teeth, dental implants do not display comparable periodontally associated bone structures like bundle bone (Lang & Lindhe, 2015). This potentially leads to a different post‐explantation behavior of the remaining defect as compared with post‐extraction sockets, because no accentuated resorption of a bundle bone can be assumed. Till to date, however, there is—according to the best author's knowledge—no evidence in the literature existing, which analyzed the amount of expectable bone remodeling or resorption after removal of osseointegrated but failing implants.

As mentioned above, peri‐implantitis is still the main reason for late implant failure causing a removal (Anitua et al., 2017). Therefore, inflammatory degradation processes may cause respective bone loss and bony defects. Schwarz et al. (2007) assessed the configuration of peri‐implantitis defects in human and dogs. As assessed by open flap surgery, mainly two different types of defects were identified (Petersen et al., 2005): well‐defined intrabony defects (Class I defects) and (Buser et al., 2012) horizontal bone loss (Class II defects). Class I defects could be further subdivided in five groups by the frequency of occurrence (a–f): Circumferential defects (Class Ie), incomplete circumferential defects with vestibular dehiscence (Class Ib), dehiscence with complete circumferential defects Class Ic, circumferential defects with vestibular and oral dehiscence (Class Id) and vestibular dehiscence defect type (Class Ia), which occurred in 55.3%, 15.8%, 13.3%, 10.2%, and 5.4% of the investigated cases, respectively. The most Class I defects (c–e) defects seemed to be associated with a Class II defect, (Schwarz et al., 2007) that is, horizontal bone loss. (Schwarz et al., 2007) The defect patterns were also hypothetically related to previous augmentation procedures, which might have influenced the remaining architecture. Therefore, most of these defects require some additional bone augmentation procedures if implants are intended to be placed immediately or after a short time of healing in a previously failed site. Because circumferential defects behave like three‐wall defects and are self‐containing, they may obviously offer the best outcome for GBR (Guided Bone Regeneration) procedures.

Because the implant bed configuration is of outmost importance to a stable implant placement, the expected morphology of the remaining defect should also guide the choice over the method of the implant removal. Whereas Ia defects might be more difficult to remove with some techniques, deep Id defects allow for a faster and easier removal—maybe even with a forceps or lever. However, respective data on that aspect are inexistent or scarce.

Especially for patients with bisphosphonate or other drugs affecting the bone metabolism, as in any surgery involving bone, care has to be taken and the least possible trauma to bone tissues has to be guaranteed. More research on that topic is also warranted.

## 8 QUESTION 5: WHAT SHOULD BE CONSIDERED WHEN REINSERTING IMPLANTS?

Gomes et al. (2018) recently conducted a meta‐analysis including 11 articles. Two additional papers, which we could identify and which were published in the meantime, are included in Table 2 (Anitua et al., 2017; Raghoebar et al., 2018). In summary, 704 replaced implants in 579 patients were analyzed. A survival rate of 88.7% for implants in previous failed sites and 85% in sites with a second time failed implant were described.

Throughout the studies, implant survival was determined as an implant still in position, whereas success in addition included the absence of peri‐implant inflammation and an aesthetically satisfied patient.

Different numbers of patients were included, ranging from only nine (Covani et al., 2006) to 144 participants (Mardinger et al., 2012).

Taking a closer look at the included studies, it is important to determine the reasons for implant failures: Different investigations assessed the reasons for implant failures: Again, mechanical fractures (Covani et al., 2006) or peri‐implantitis were found, whereas others did not differentiate and had patient cohorts with several different causes for implant failure (Machtei et al., 2008; Machtei et al., 2011; Manor et al., 2015; Mardinger et al., 2012; Quaranta et al., 2014; Wang et al., 2015). Rarely, multiple implant failures were observed in one patient simultaneously. However, this has been described in the literature under the name “cluster effect” (Jemt & Johansson, 2006). Usually, this phenomenon occurs soon after implant placement, (Roos‐Jansåker, Lindahl, Renvert, & Renvert, 2006) which indicates that systemically or genetically modifying implications may exist, which should be controlled before reperforming any new additional implant surgery (Greenstein & Cavallaro, 2014).

Implant survival of a second implantation after failures was shown to reach an overall success rate of 71–100% after 7 to 180 months. In a third attempt, the rate is described as of 50–100% after 7 to 180 months. Implant success, however, was only described by few authors ranging from 90 to 100% (Table 2).

Wang et al. (2015) assessed the survival and success rates of implant replacement after early failure (failure in attaining osseointegration). The results accounted for 94.6% (survival) and 90.6% (success rate) after an average of 69.4 months and did not significantly differ from results obtained after late implant failure. The lower survival rates in some studies may be partially explained by the diameters and lengths of the implants chosen as replacement for the previous failing implant. If implants were described as being similar in diameter and shorter in length, it resulted already in a lower survival rate (Chrcanovic et al., 2017; Grossmann & Levin, 2007). After removal of an implant, a defect is created with at least the size of the failing implant. Thus, considerations regarding the primary stability of the replacement implant have to be taken. The successor implant should be chosen in a larger size or the defect should be a subject to grafting or healing and a second stage implant placement. The authors therefore concluded that especially implants with rough surfaces and wider diameters may be preferred or considered as an advantage for the overall survival and success rate (Chrcanovic et al., 2017; Grossmann & Levin, 2007). Especially in the context of immediate replacement procedures, implants should therefore be of larger size in order to gain adequate primary stability (Chrcanovic et al., 2017; Grossmann & Levin, 2007).

Other authors also evaluated the role of the implant surface on the survival. They corroborated the idea that rough surfaces with a larger bone‐implant interface seem more favorable and lead to a statistically significantly better results (Alsaadi et al., 2006; Chrcanovic et al., 2017).

Replacement of failing implants was also described in sinus‐grafted sites: Whereas the initially inserted implants predominantly failed due to lack of osseointegration (77.3%), second implant surgery led to 100% and 92% survival rates in additionally grafted and nongrafted sites, respectively. In conclusion, authors conclude that sinus grafting is no obstacle for a second attempt implant surgery (Manor et al., 2015).

So far, no evidence exists in literature, which might pinpoint any statistical difference in outcomes when comparing immediately or late performed “reimplantations.” Therefore, given the premise of sufficient bone quantity and quality in the absence of infection after explantation, a new implant can be placed in the same session (Kim et al., 2010). In contrast, if the explantation leads to more extended bone defects, a correction of the buccal bone plate is mostly needed in a first step using GBR procedures (Anitua et al., 2017). Additional research on these aspects are required as well.

## 9 CONCLUSIONS

Regarding the removal of dental implants, limited data are still available in the literature: Evidence is still based mainly on case reports or on studies with a limited number of patients.

Based on this narrative review, the following conclusions can be drawn:

- Early and late implant failure normally have different causes and can be treated in different ways.
- Peri‐implantitis shows to be the main reason for late implant failure.
- Trephine burs seem to be the best‐known method for implant removal.
- The CTRT method alone or combined, because of the low invasiveness, should be the first choice for the clinician.
- Scarce data regarding zirconia implant removal are available. Because of zirconia's physical properties, it is supposed that they require a different approach compared with titanium implants.
- The defect type of the bone at the failed implant is crucial for the choice of the removal method and the subsequent treatment.
- Implantation in previously failed sites irrespective of an early or late failure results in 71% to 100% survival after an average of 69.4 months.

**Figure 4:**
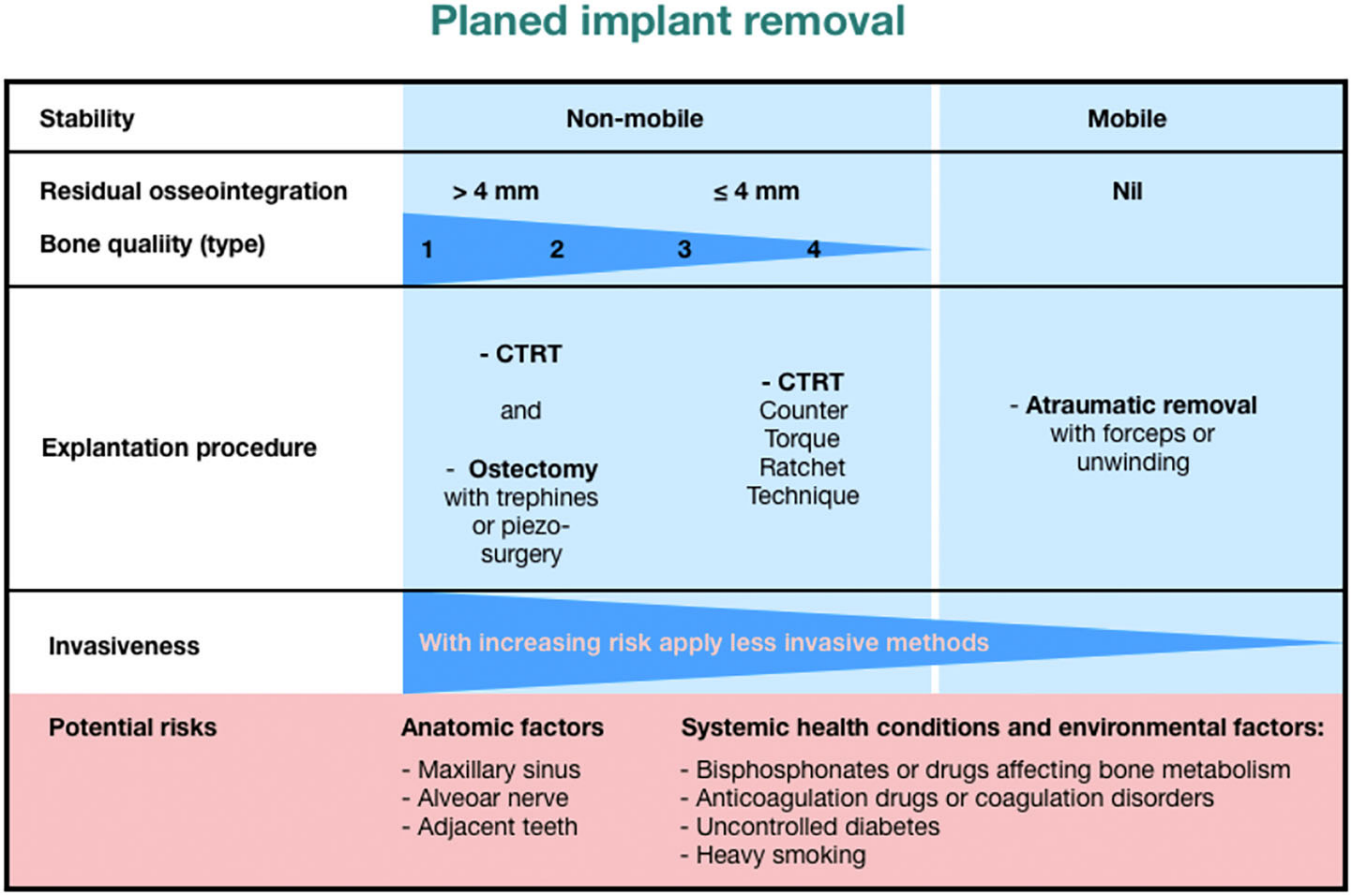
Clinical recommendations

Figure 4 shows some suggested basic clinical recommendations by the authors.

## CONFLICT OF INTEREST

The authors declare no conflict of interest.

## FUNDING INFORMATION

The work was supported by the Clinic of Preventive Dentistry, Periodontology and Cariology, Center of Dental Medicine, University of Zurich.

## COMPLIANCE WITH ETHICAL STANDARDS

## ETHICAL APPROVAL

This article does not contain any studies with human participants or animals performed by any of the authors.

## INFORMED CONSENT

For this type of study, formal consent is not required.
